# A New Cone for Root Canal Filling

**Published:** 1901-04-15

**Authors:** Theo. F. Von Beust

**Affiliations:** Dresden, Germany


### A NEW CONE FOR ROOT CANAL FILLING.

BY THEO. F. VON BEUST, M.D., D.D.S., DRESDEN, GERMANY.

Reprinted from *Items of Interest.*

To the many methods of filling root canals now mentioned in our dental journals, I venture to add another one. It advances neither a departure from the methods, the materials nor from the preparatory treatment of root canals now in vogue, but distinguishes itself from all other fillings that have come to my notice, in that the canal is more easily and completely filled, and in that it is removable, a property which itself must make it invaluable to every dentist. I have had opportunities of testing this filling in every respect, and can say that it can be easily and completely removed the moment the contents of the pulp chamber are exposed to the reach of an excavator or pair of light pliers. It therefore also asserts itself as a practical test filling, and can be used in the antiseptic treatment of roots.

My method is this: I take fine silver wire of from 0.05 to 0.02 millimeter in diameter, and cut from the different thicknesses of wire lengths to correspond to the different sizes of root canals. These I roughen and surround with a film of gutta-percha, making the point cylindrical or cone-shaped, very like the root canal points bought at the depots. Then I turn a crook or hook at that end of the point which is to occupy the coronal portion of the pulp cavity. Thus we have an ordinary root canal point of gutta-percha with a core of wire and a hook at the larger end.

This hook facilitates the removal of the filling, it being readily seen that the moment one can reach it with the pliers, the filling can be drawn bodily from the canal.

Removability has been the *bete noir* of most of our practitioners, and I trust that this little idea may prove of value to some of the gentlemen of the profession. It has, moreover, other advantages, in that the filling, which can be made as fine as any practical gutta-percha point, has such strength that it will follow the root canal to the very end without turning at the point, as is the case with the gutta-percha points.

My method of filling is to introduce chloro-percha into the root, and then insert the point with a pumping motion, so as to better force out all air that may be confined. The completed filling, it will thus be seen, has exactly the mechanical and therapeutic value of an ordinary gutta-percha filling, with the additional advantages above named.

I am well aware that Dr. Morrison and Dr. Francis Peabody, of Louisville, my dear old teacher, used metal points in connection with chloro-percha, but the use of a ready-made gutta-percha point, with a core of wire as here explained is, as far as I know, new.

